# Metacarpal Titanium Elastic Nail insertion

**DOI:** 10.1308/003588412X13373405387050c

**Published:** 2012-10

**Authors:** A Keightley, A Hacker

**Affiliations:** Ashford and St Peter’s Hospitals NHS Foundation Trust,UK

**Figure 1:**
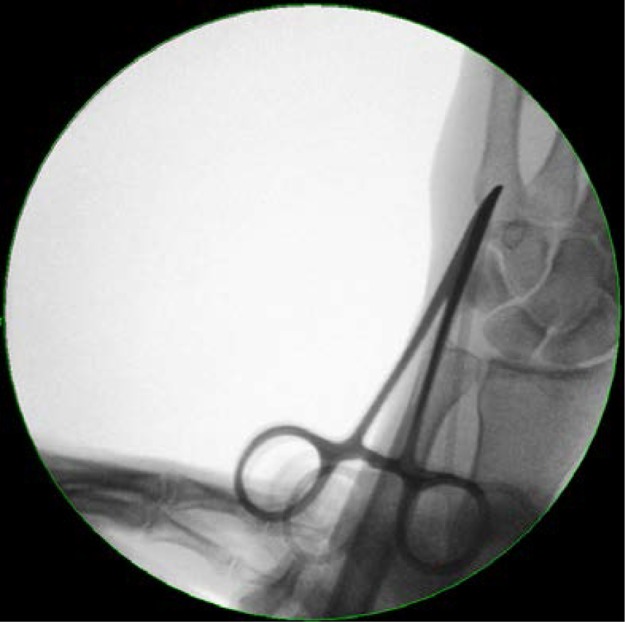
Entry point gained using clip to penetrate the near cortex

**Figure 2:**
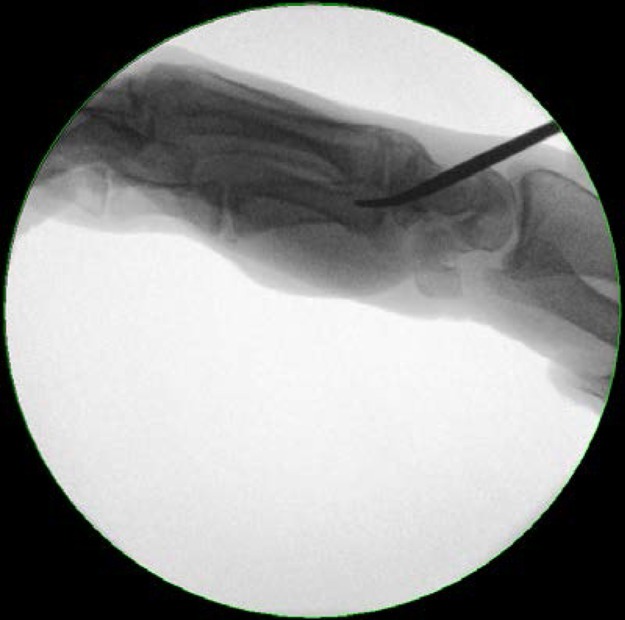
Clip turned 180° to continue along medullary canal

**Figure 3:**
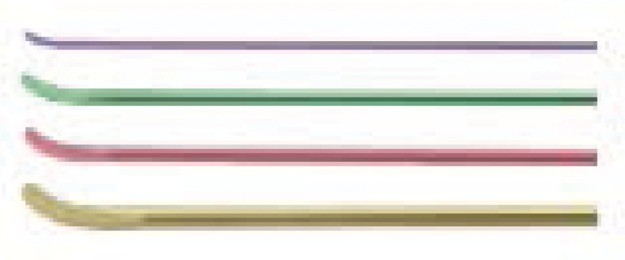
Titanium Elastic Nails, showing the curved profile

Standard Titanium Elastic Nail (TEN; Synthes, Welwyn Garden City, UK) fixation requires a 2.5mm drilled entry point at the base of the metacarpal. This can damage soft tissues including the extensor tendon and also risks breaching the volar cortex. We recommend opening the dorsal metacarpal cortex with a curved artery clip (Fig 1). Initially, point the tip downwards to penetrate the cortex, turning the clip 180° to advance it down the medullary canal (Fig 2). The curve on the clip mimics the profile of a 2.0mm TEN (Fig 3) and its natural entry to the metacarpal. The less traumatic nature of this approach may lead to improved soft tissue protection.

